# Computed tomography coronary angiography is the way forward for evaluation of children with Kawasaki disease

**DOI:** 10.21542/gcsp.2017.28

**Published:** 2017-10-31

**Authors:** Manphool Singhal, Pankaj Gupta, Surjit Singh, Niranjan Khandelwal

**Affiliations:** Advanced Pediatrics Center, Post Graduate Institute of Medical Education and Research (PGIMER), Chandigarh, INDIA-160012

## Abstract

Kawasaki disease (KD) is an acute idiopathic vasculitis affecting infants and children. Coronary artery abnormalities and myocarditis are the major cardiovascular complications of KD. Coronary artery abnormalities develop in 15–25% of untreated KD. Two-dimensional transthoracic echocardiography has hitherto been considered the modality of choice for evaluation of children with KD. There are, however, several limitations inherent to echocardiography - including limited evaluation of distal vessels, left circumflex artery and poor acoustic window in growing children. Catheter angiography is the gold standard for evaluation of coronary artery abnormalities in older children and adults; however it also has inherent limitations - including complications related to its invasive nature, higher radiation exposure, and inability to evaluate intramural abnormalities. Thus serial invasive coronary angiography studies are not feasible in children. There have been major advances in computed tomography (CT) coronary imaging so that it is now possible to delineate the coronary artery anatomy with higher temporal resolution and motion-free images at all heart rates with acceptable radiation exposure. There is, however, a paucity of literature with regard to the use of this technique in children with KD. In this review, we discuss the application of computed tomography coronary angiography (CTCA) in children with KD with special reference to strategies aimed at reducing the effective radiation dose.

## Introduction

KD is a vasculitic disorder in children with a predilection for coronary arteries. Without appropriate treatment, up to 25% children with KD can develop coronary artery abnormalities (CAA). With early diagnosis and prompt therapy the complications can be reduced to 3–5%. Two-dimensional echocardiography is the imaging modality of choice for assessment of coronary arteries during the acute stage and on follow-up^1^. Echocardiography, however, has its own inherent limitations. It is highly operator dependent, requires experience and may not be able to delineate distal segments of coronary arteries and left circumflex artery. Further, as the child grows, evaluation of coronary arteries becomes difficult because of a limited acoustic window^1, 2^. Other imaging techniques can supplement echocardiography. However, there are no clear-cut recommendations or guidelines on their usage in children with KD. Catheter angiography is an option but it is invasive, cannot be repeated at frequent intervals, requires sedation and is associated with high radiation exposure. It may also fail to detect intramural changes in coronary arteries^3^. Magnetic resonance (MR) coronary angiography is technically difficult and time consuming and few centres have the requisite expertise in performing the procedure in young children^1^. With significant advancements in CT technology including the availability of increased detector rows and advanced software, CTCA is expected to allow a comprehensive non-invasive evaluation including luminal changes, intramural changes and plaque morphology objectively along the entire course of coronary arteries at reasonably low radiation dose^4^. Thus CTCA is an alternative imaging modality for non-invasive diagnosis and follow-up of children with KD. Accordingly, its incorporation into the management algorithm of KD should be considered.

## Coronary artery lesions in KD

With early diagnosis and appropriate management, there has been a dramatic reduction in occurrence of CAA in children with KD. However, approximately 3–5% of treated children still go on to develop CAA^5, 6^. These CAA can evolve into stenotic lesions or occlusions. CAA may decrease in size during the convalescence phase^4^. However, remodelling of CAA occurs in only half of these patients over the first 2 years of follow-up^6, 7^. All of these children may develop significant residual disease and complications in adulthood. This serves as the basis for long term cardiovascular follow-up of patients with KD. Coronary stenosis may be segmental or localized. Segmental stenosis refers to neovascularisation and recanalization in a previously occluded segment^8^. This complication is encountered in 15% patients and involves the right coronary artery most frequently. Localised stenosis of greater than 75% develops in 5–12% patients in the second decade following disease onset^9^. It has a predilection for proximal trunk of left anterior descending.

## Imaging in follow up of KD

CAAs in KD require long-term follow-up. The aim is to determine the progression of the CAA and monitor development of thrombotic and steno-occlusive complications. The American Heart Association (AHA) proposes the application of interval echocardiography, stress echocardiography, and invasive or non-invasive coronary angiography in follow up of patients with KD^1^.

**Figure 1.**
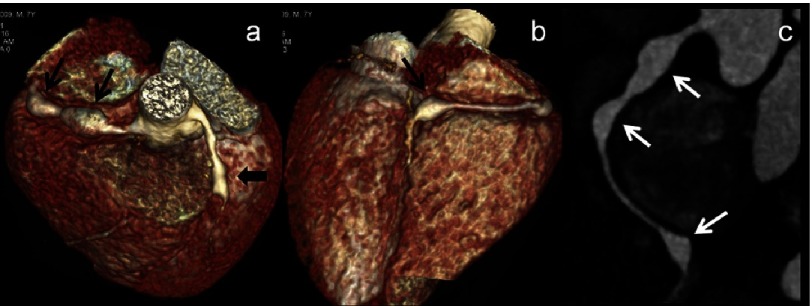
(a–c): CT Coronary angiography (CTCA) images (a & b - volume rendered images-VRT) and (c) curved maximum intensity projection (MIP) images of a 7 year male child in acute phase show two fusiform aneurysms in proximal right coronary artery (RCA) and one in distal RCA at its bifurcation into its posterior descending and postero-lateral branches (thin arrows). Note fusiform aneurysm in mid left anterior descending artery (LAD) as well (a-thick arrow). 2D-transthoracic echocardiography could detect one aneurysm in proximal RCA and proximal LAD and missed two aneurysms in RCA.

**Figure 2.**
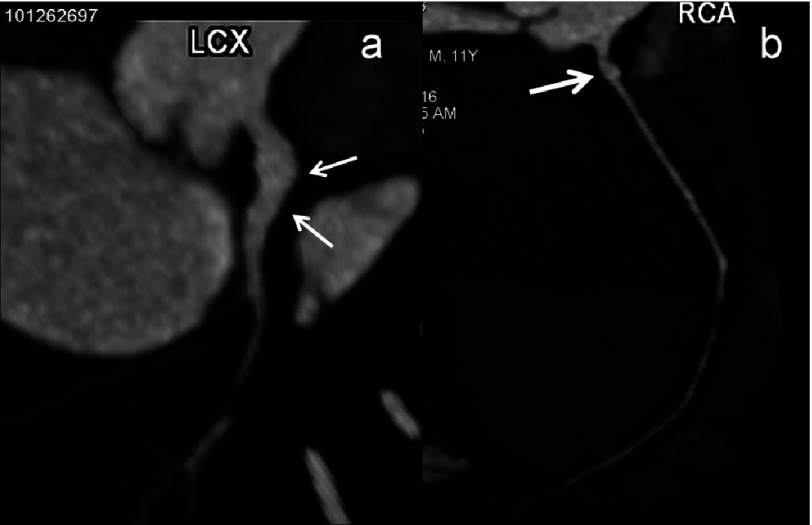
(a–b): CTCA curved maximum intensity projection (MIP) images of a 11 year male child in convalescent phase (5 years after diagnosis) show fusiform aneurysmal dilation (a - thin arrows) of proximal left circumflex artery (LCX) and saccular aneurysm of proximal RCA (thick arrow). Left circumflex artery is usually not well evaluated on 2D- transthoracic echocardiography.

**Echocardiography** is the imaging technique of choice for follow up^6^. Limitations of echocardiography include operator dependence and poor delineation of distal segments of the coronary arteries and left circumflex artery^1^ (Figures 1 and 2). Besides, it has poor sensitivity in demonstrating intra-luminal thrombus or arterial stenosis^1^. Furthermore, as the child grows, assessment of coronary arteries becomes more difficult. Nevertheless, the preliminary examination performed in the acute phase serves as a baseline for longitudinal follow-up.

**Invasive catheter angiography** is considered the gold standard for evaluation of coronary arteries. However, it is an invasive procedure and is associated with significant complications. Its routine use in serial follow up of children with KD is also limited by the associated radiation hazard. Typical dose in diagnostic catheter angiography is 5-7 mSv^10^. Though, catheter angiography is exquisitely sensitive to intraluminal changes, it does not specifically detect intramural changes. Post-mortem studies of patients with KD in whom CAA were shown to regress with catheter angiography revealed intimal proliferation and fibrosis not detected on angiogram^11^. As mural changes are an important component of cardiovascular complications in KD, an imaging modality that is capable of evaluating the lumen as well as the wall of coronary arteries is necessary.

## CT coronary angiography

### Background and evolution

With the early generations of CT scanners, sensitivity for grading native coronary disease was low. However with advent of 64-slice CT, gantry rotation time was reduced to 330 ms leading to marked improvement in temporal resolution for cardiac electrocardiography (ECG)-gated imaging^12^. This translated to improved image quality and high sensitivity and specificity in the evaluation of all coronary artery branches and segments. Increase in the number of acquired slices to 128, 256 and 320 and resultant increase in z-axis coverage has further improved the temporal resolution^13^. With 320-slice CT, cranio-caudal coverage of 16 cm allows demonstration of the entire coronary arteries in a single heartbeat^14^. With dual-source CT (DSCT), the temporal resolution is improved to 73 ms. This reduces the need to control the heart rate during the scan. Studies comparing the diagnostic performance of DSCT and single-source CT in the evaluation of coronary arteries of a wide range of patient subsets (including those with atrial fibrillation) demonstrated high diagnostic accuracy for DSCT^14^. This is particularly vital in children who cannot be administered large doses of beta-blockers.

### Rationale for use of CTCA in KD

Changes in coronary circulation in KD affect the lumen as well as the wall. In this context, CTCA not only has the capability to depict changes in luminal calibre, it can also evaluate intramural changes including plaque characterisation. The current generation of CT scanners have a spatial and temporal resolution that allows the evaluation of all coronary artery branches and segments with exquisite detail. Thus, CTCA is an ideal non-invasive imaging modality for evaluation of CAA in KD.

### Challenges: radiation issues

The prime concern in the use of CTCA in children is the radiation hazard. Although CT constitutes only 15% of all radiological investigations, it entails high radiation dose and accounts for up to 70% radiation dose from diagnostic radiological procedures^15^. This becomes highly relevant in view of the recent reports of radiation induced malignancy. It is estimated that a radiation exposure of 10 mSv will result in development of malignancy in 1 in 2000 population exposed^16^. In a study by Brenner and Hall, 1.5–2% of all cancers in the USA were attributed to radiation exposure from CT scans^15^. Thus an appropriate balance between radiation dose and image quality must be reached. Concerns over escalating radiation doses have led to various strategies being adopted for dose reduction in CTCA. Fortunately, all of the latest generation scanners (at least 256 detector rows) provide better image quality and lower patient dose than earlier generation scanners. Of all the dose-saving strategies in CTCA including child-size bowtie filter, body size-adapted protocol including low tube voltage techniques; ECG-controlled and attenuation-based tube current modulations, and prospectively ECG-triggered sequential scanning, the last approach is the most critical and is discussed below in detail.

## Prospective ECG triggering

Prospective ECG triggering is based on the premise that CT data is acquired only during the particular cardiac phase by stimulating the X-ray tube only when signalled by the ECG signal. In rest of the R–R interval, the X-ray tube current is switched off or markedly lowered. A lowering of effective dose up to 90% compared to retrospective ECG-gating has been reported in studies using single-source 64-slice or DSCT^17–19^. This comes at a diagnostic value similar to that of CTCA with retrospective ECG-gating. With application of prospective ECG triggering, a radiation dose comparable to catheter angiography has been reported. The condition for successful application of this technique is a low and regular heart rate. Thus, heart rate control with pharmacological measures is required for prospective gating. However, with the advent of DSCT, the heart rate control for prospective gating has been rendered less strict. Misalignment due to acquisition of images in more than one heartbeat is another potential limitation of prospective gating^17^. This is partly overcome by the latest 320-slice CT, which allows the entire heart volume to be covered in a single heartbeat.

A study by Duan et al. in 19 children with KD reported the mean CT doses of prospective ECG-triggered CTCA as low as 0.4 mSv^19^. Thus it can be seen that recent CTCA technology has led to acceptable CT radiation dose. In a study assessing the doses from various pediatric protocols in infants using a phantom, effective doses of *1.49* ± *0.10* and *4.66* ± *0.40* mSv for non-gated and retrospectively gated scans, respectively was reported in a 64-slice CT scanner^20^. In a 256-slice CT, effective doses were *2.15* ± *0.15*, *6.87* ± *0.56* and *1.12* ± *0.11* mSv for non-gated, retrospectively gated and prospectively gated scans, respectively^20^. The authors concluded that prospectively gated CTCA protocol allows good quality images with relatively low dose. Prospectively gated CTCA protocol in fact had better image quality than the retrospectively gated scan with 83.70% dose reduction^20^.

Huang et al. conducted a study to assess the diagnostic performance of prospectively gated 256-slice CTCA in infants^21^. They reported that diagnostic quality images were possible in all cases with diagnostic accuracy up to 95.9%.

Duan et al. investigated the role of prospective ECG-triggered dual-source CT coronary angiography in 19 infants and children with KD and coronary artery aneurysms^19^. They found that the image quality and inter-observer agreement were excellent. The mean effective dose was 0.36 ± 0.06 mSv.

In a study by Kim et al., five children below the age of 2 years, a radiation dose of only *0.6* ± *0.5* mSv (range, *0.2–1.3* mSv) was reported using prospective ECG-triggered sequential CT scanning^22^. Our own experience with prospective ECG-triggering is in line with the results of studies that support its routine use in pediatric CTCA^23, 24^.

Using the dose saving strategies discussed above, doses less than 1 mSv have been reported in the literature^25^. This can be achieved with a combination of strategies including prospective ECG triggering. With such a low radiation dose, widespread application of CTCA in children in general and those with KD in particular should become routine.

## CT coronary angiography: comparison with echocardiography

**Figure 3.**
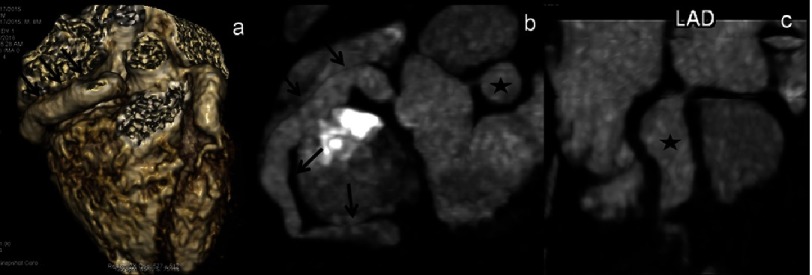
(a–c): CTCA VRT (a) and curved (MIP) images (b & c) of an 8 year male child in acute phase show aneurysmal dilation of entire RCA (a & b - thin arrows) and a giant aneurysm in proximal LAD (b & c - asterisk). 2D-transthoracic echocardiography detected aneurysm in both the arteries; however it failed to delineate the extent of involvement of RCA.

**Figure 4.**
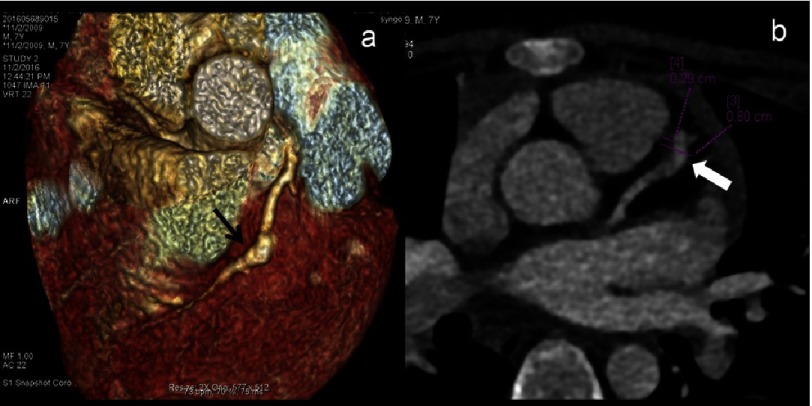
(a–b): CTCA VRT (a) axial (b) images of a 7 year male child in convalescent phase (4 years after diagnosis) show fusiform aneurysmal dilation (a - thin arrows) of proximal LAD; (b) shows a giant aneurysm with eccentric thrombus along the anterior wall (thick arrow). 2D-transthoracic echocardiography though could detect aneurysm but thrombus was missed.

All the segments of the main coronary arteries may be involved in KD, although the lesions are most often proximal. It is often not appreciated that echocardiography has only a limited role in evaluation of middle and distal coronary arteries and left circumflex artery^26^. CTCA provides an ideal method for evaluation of all the segments of the coronary artery (Figures 3 and 4).

In a study of 24 children with KD, the efficacy of echocardiography was compared with CTCA in evaluation of CAA^26^. Echocardiography failed to detect 8 small aneurysms located in mid and distal segments [LAD2 (*n* = 1), LCX (*n* = 2), RCA2/3 (*n* = 4) and D1 (*n* = 1)]. CTCA also detected a stenosis of LAD1 that was missed on echocardiography.

Chu et al. reported failure of echocardiography to detect the distal lesions in a study comprising 8 children with KD^27^. Echocardiography missed 3 of the 12 aneurysms located in RCA3 (*n* = 1) and LAD1 (*n* = 2).

In the study by Xing et al. 4 aneurysms were missed in the LCA (*n* = 3) and LAD (*n* = 1) on 2D-echocardiography. In addition, stenosis and calcification were missed in the LCA (*n* = 3)^28^.

Peng et al. reported the comparison of CTCA and 2D-echocardiography in 12 boys with KD. Echocardiography missed 8 of the 30 aneurysms detected by CTCA^29^. These lesions were location in the LAD (*n* = 2), LCX (*n* = 1), and RCA (*n* = 5). In addition, echocardiography missed calcification and stenotic lesions.

In the study by Duan et al., echocardiography failed to detect 7 aneurysms^19^. In all the reported studies, there was a good correlation between echocardiography and CTCA for the size of the detected aneurysms.

## CTCA: comparison with MR coronary angiography (MRCA)

**Figure 5.**
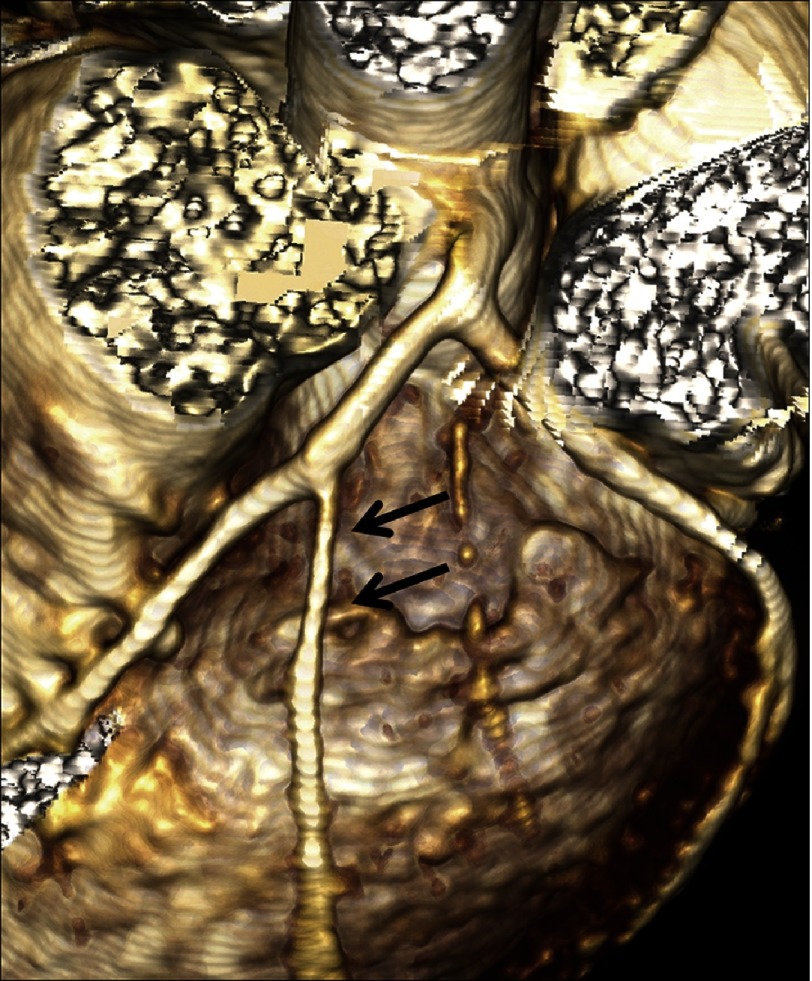
CTCA VRT 8 year female child in convalescent phase (5 years after diagnosis) show long segment stenosis of diagonal branch of LAD (arrows). 2D-transthoracic echocardiography has limitation to detect coronary artery abnormalities of coronary artery branches.

MRCA has also been evaluated as one of the non-invasive imaging modalities in patients with KD. In a usual clinical setting, one has to choose between MRCA and CTCA as the best imaging modality. Studies in a small cohort of patients with KD, having only aneurysms, have reported good agreement between MRCA and CCA^30, 31^. However, others have reported lower diagnostic performance of MRCA^32, 33^. This can be attributed to lower spatial and temporal resolution and poorer image quality compared to CTCA^34^. The longer scan time of MRCA in young children is particularly detrimental to the technical success rate of MRCA^35^. Studies on patients with steno-occlusive lesions reported a greater accuracy of CTCA over MRCA (Figure 5). Situations in which MRCA is preferred over CTCA include evaluation of coronary arteries with heavy intra-mural calcifications (leading to blooming artifact on CT) and assessment of myocardial perfusion, viability, and fibrosis^36^.

## CTCA: comparison with catheter angiography

Catheter angiography is an invasive procedure and requires hospital admission for duration dependant on institutional protocols. There is a definite risk of minor and major complications. Although, it has the highest spatial resolution that although evaluation of the coronary artery lumen with a high degree of accuracy, it fails in evaluation of the coronary artery wall. CTCA scores over catheter angiography in evaluation of intraluminal thrombus, wall thickening and calcification. There are only a few studies prospectively comparing the performance of catheter angiography and CTCA in children with KD but the advantages of CTCA are clearly demonstrated by its utilisation in other conditions. In a study by Tsujii et al., the accuracy of CTCA and catheter angiography in measurement of coronary artery aneurysms was compared^37^. Twenty-two children with coronary artery lesions were retrospectively evaluated. There was excellent agreement between CTCA and catheter angiography in measurement of aneurysms.

## Conclusion

CTCA is a robust imaging modality in evaluation of children with KD. With studies reporting CT doses less than 1 mSv, it is expected to be used more frequently both in the acute and chronic phases of the disease. In near future, CTCA may be incorporated in the management algorithm of children with KD.

## ABBREVIATIONS

CTCA: Computed tomography coronary angiography
CAA: Coronary arterial abnormalities
CTDI vol: CT dose index
DLP: Dose length product
DSCT: Dual source computed tomography
ECG: Electrocardiogram
KD: Kawasaki disease
MR: Magnetic resonance
